# Circulating Tumor DNA Across the Patient Journey in Pancreatic Cancer: From Molecular Profiling to Dynamic Disease Monitoring

**DOI:** 10.3390/cancers18213471

**Published:** 2026-10-27

**Authors:** Makoto Abue, Naoki Asano, Takanori Morikawa, Atsushi Masamune, Satoshi Nishizuka, Jun Yasuda

**Affiliations:** 1 Department of Gastroenterology, Miyagi Cancer Center, Natori 981-1293, Miyagi, Japan; 2 Division of Cancer Stem Cell, Miyagi Cancer Center Research Institute, Natori 981-1293, Miyagi, Japan; 3 Division of Carcinogenesis and Senescence Biology, Tohoku University Graduate School of Medicine, Sendai 980-8575, Miyagi, Japan; 4 Department of Gastroenterological Surgery, Miyagi Cancer Center, Natori 981-1293, Miyagi, Japan; 5 Division of Gastroenterology, Tohoku University Graduate School of Medicine, Sendai 980-8574, Miyagi, Japan; 6 Department of Gastrointestinal Aging Control, Healthspan Research Center, Tohoku University, Sendai 980-8575, Miyagi, Japan; 7 Division of Biomedical Research & Development, Iwate Medical University Institute for Biomedical Sciences, Yahaba 028-3694, Iwate, Japan; 8 Division of Molecular and Cellular Oncology, Miyagi Cancer Center Research Institute, Natori 981-1293, Miyagi, Japan; 9 Division of Cancer Molecular Biology, Tohoku University Graduate School of Medicine, Sendai 980-8575, Miyagi, Japan

**Keywords:** pancreatic ductal adenocarcinoma, circulating tumor DNA, liquid biopsy, molecular residual disease, molecular recurrence, treatment monitoring, precision oncology

## Abstract

Background/Objectives: Circulating tumor DNA (ctDNA) provides molecular information at different stages of the pancreatic ductal adenocarcinoma (PDAC) patient journey, but its clinical value differs according to when and why it is measured. This narrative review examines ctDNA from diagnosis and molecular profiling through neoadjuvant therapy, postoperative molecular residual disease (MRD), surveillance, and dynamic monitoring during systemic therapy, critically assessing the strength and limitations of the evidence at each clinical stage and its implications for clinical decision-making. Methods: Relevant studies were identified primarily through PubMed/MEDLINE, supplemented by forward citation tracking in Google Scholar, backward reference screening, and ClinicalTrials.gov searches for ongoing interventional studies. Original PDAC clinical studies were prioritized, with selected reviews, guidelines, consensus statements, and position papers used for context. Results: In localized disease, limited ctDNA shedding constrains diagnostic sensitivity, although baseline detection may provide prognostic information. After surgery, ctDNA positivity consistently identifies patients at increased risk of recurrence, whereas a negative result cannot reliably exclude residual disease. During surveillance, newly detectable or rising ctDNA may precede radiological recurrence. In advanced disease, serial ctDNA kinetics provide increasingly consistent information on treatment response and progression. Evidence for dynamic monitoring is increasing, but evidence that ctDNA-guided treatment or surveillance improves patient outcomes remains insufficient. Conclusions: The clinical value of ctDNA depends on when and why it is measured. ctDNA should complement rather than replace conventional assessments. By distinguishing established prognostic associations from emerging monitoring applications and unproven ctDNA-guided interventions, this review provides a practical framework for interpreting ctDNA across the PDAC patient journey. Prospective trials are needed to determine whether ctDNA-guided decisions improve patient outcomes.

## Simple Summary

Circulating tumor DNA (ctDNA) provides molecular information from a blood sample, but its clinical value in pancreatic cancer changes across the patient journey. Before treatment, ctDNA may complement tissue-based molecular profiling and help refine risk assessment, although limited release of tumor DNA into the bloodstream reduces sensitivity in localized disease. After surgery, detectable ctDNA can identify patients at high risk of recurrence, and serial testing during follow-up may reveal molecular recurrence before it becomes visible on imaging. During treatment for advanced disease, changes in ctDNA may provide an early indication of response or progression. ctDNA should therefore complement, rather than replace, tissue analysis, imaging, CA19-9, and clinical assessment. The key remaining question is whether using ctDNA results to guide treatment or surveillance improves patient outcomes.

## 1. Introduction

Management of pancreatic ductal adenocarcinoma (PDAC) requires accurate assessment of disease extent, treatment response, and recurrence at different stages of care [1,2]. Anatomical imaging is indispensable for diagnosis, staging, and response assessment, but radiographic changes may not fully reflect pathological treatment response, particularly after neoadjuvant therapy [3,4]. Serum carbohydrate antigen 19-9 (CA19-9) is clinically useful but is limited by Lewis antigen status and by non-malignant conditions including biliary obstruction and inflammation [5,6]. These limitations support the need for additional biomarkers that can complement imaging, CA19-9, and pathological assessment.

Circulating tumor DNA (ctDNA), the tumor-derived fraction of cell-free DNA (cfDNA), provides molecular information from a minimally invasive blood sample. Its short circulating half-life creates the possibility of near-real-time assessment of changes in tumor burden and biology [7,8]. In PDAC, the high prevalence of KRAS mutations [9,10] has provided a practical molecular target for KRAS-focused ctDNA assays [11]. Broader sequencing approaches have further enabled detection of additional genomic alterations in plasma [12].

As ctDNA assays have improved, the clinical question has also changed. The question is no longer only whether ctDNA can be detected, but how the result should be interpreted at each stage of the patient journey. Yet sensitivity remains strongly dependent on disease burden, tumor shedding, assay design, and sampling context [13]. Analytical performance alone does not establish clinical utility.

Previous reviews and consensus statements have discussed the analytical and clinical requirements for ctDNA testing and its potential roles across diagnosis, prognosis, treatment monitoring, and follow-up, including the distinction between clinical validity and clinical utility [14,15]. Building on this foundation, a patient-journey perspective can help clarify how the strength and clinical implications of ctDNA evidence differ across disease settings [16].

**Figure 1:**
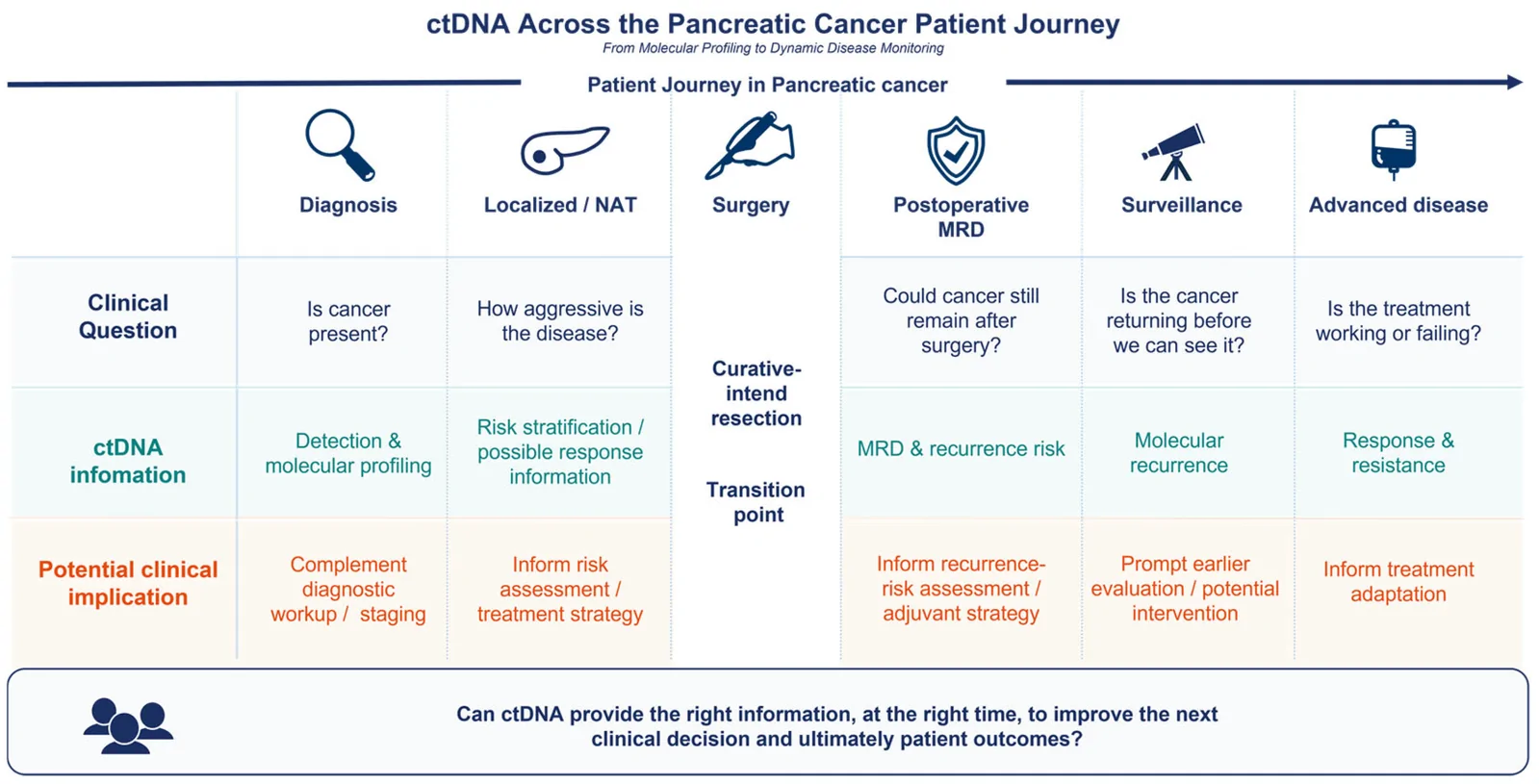
ctDNA across the patient journey in pancreatic cancer. Schematic overview linking major clinical stages with the questions ctDNA may help address and the decisions it may eventually inform. ctDNA is presented as complementary to imaging, tissue analysis, CA19-9, pathology, clinical factors, and patient preferences. ctDNA, circulating tumor DNA; NAT, neoadjuvant therapy; MRD, molecular residual disease.

This structured narrative review therefore examines ctDNA across the PDAC patient journey, from diagnosis and molecular profiling through neoadjuvant therapy, postoperative molecular residual disease (MRD) and surveillance, to dynamic monitoring during systemic therapy. We focus on what a positive or negative ctDNA result means at each stage of the patient journey, what it adds to conventional assessment, and whether the available evidence is sufficient to change patient management. The overall clinical framework is summarized in Figure 1.

## 2. Methods

This narrative review was based primarily on literature identified through PubMed/MEDLINE. Searches were supplemented by forward citation tracking in Google Scholar and backward screening of relevant references. Original clinical studies were prioritized, particularly those addressing ctDNA across key stages of the PDAC patient journey, while selected reviews, guidelines, consensus statements, and position papers were used to provide broader context. Studies were selected based on their relevance to the clinical questions addressed in this review, with particular attention to study population, assay methodology, and clinical outcomes. Ongoing interventional studies evaluating ctDNA-informed management were identified through ClinicalTrials.gov. The literature search was last updated in October 2026.

## 3. Biological and Analytical Framework

ctDNA constitutes a variable and often small fraction of total circulating cfDNA [17]. Its detectability varies with disease extent, with lower detection rates generally observed in localized than in metastatic cancers [18]. In PDAC, plasma KRAS mutations are detected less frequently in localized than in advanced disease [19]. PDAC is characterized by abundant desmoplastic stroma and relatively sparse vascularization [20], and these features may contribute to limited ctDNA release into circulation, although their direct effects on ctDNA detectability remain unclear [13]. In previously treated PDAC, metastatic distribution also influences ctDNA detectability, with higher KRAS detection rates observed in patients with liver metastases than in those with metastases to other sites [21]. Therefore, a negative ctDNA result does not necessarily mean that molecular disease is absent, as too little tumor DNA may have entered the circulation to be detected [13]. The rapid clearance of circulating mutant DNA further enables ctDNA to reflect relatively short-term changes in disease burden [7].

ctDNA analysis in PDAC has evolved from predominantly mutation-focused approaches, particularly KRAS-based PCR and digital PCR, toward broader targeted next-generation sequencing (NGS) [11,13]. Contemporary assays include both tumor-naïve and personalized tumor-informed strategies [12,22,23]. These approaches differ in genomic breadth, analytical sensitivity, turnaround time, resource requirements, and dependence on available tumor tissue. Importantly, tumor-informed and tumor-naïve approaches represent different assay strategies rather than sequential generations of the same technology [24,25]. The main characteristics of these assay approaches are summarized in Supplementary Table S1.

Among mutation-focused approaches, droplet digital PCR (ddPCR) has played an important role in PDAC ctDNA research. By partitioning DNA into numerous individual reactions, ddPCR enables sensitive detection and quantitative measurement of predefined mutations, particularly KRAS alterations. Its applications in PDAC have included plasma mutation detection across disease stages [11], postoperative MRD assessment [26], and longitudinal monitoring during systemic therapy [27,28]. Broader sequencing approaches have also been used for longitudinal monitoring during chemotherapy [29]. However, ddPCR is generally limited to selected mutations, and high analytical sensitivity cannot fully overcome low ctDNA shedding, particularly in localized disease [13,18].

Pre-analytical and analytical factors are also integral to assay performance. Blood collection and processing conditions, including collection tube, processing time, centrifugation, and storage, can affect cfDNA yield and sample quality [30]. However, not all processing variations necessarily affect ctDNA measurements; for example, centrifugation protocols did not significantly alter KRAS variant allele fractions in a study involving patients with metastatic PDAC [31]. Beyond preanalytical processing, analytical factors, including sequencing depth and bioinformatic filtering, also affect assay performance [32]. Another challenge is determining where a detected mutation comes from. Somatic alterations arising from clonal hematopoiesis may be detected in plasma and incorrectly attributed to the tumor if appropriate filtering is not performed [33].

These biological and analytical limitations need to be considered when ctDNA results are interpreted clinically. The meaning of a positive or negative result depends on the clinical setting, assay, and timing of blood collection.

## 4. Diagnosis and Molecular Profiling

### 4.1. Diagnosis and Initial Assessment

ctDNA is attractive as a diagnostic biomarker because molecular information can be obtained from a blood sample without invasive tissue acquisition [11,34]. In practice, however, diagnostic sensitivity remains a major limitation, particularly in localized disease [19,34]. Early KRAS-focused studies and subsequent NGS-based analyses demonstrated that plasma ctDNA detection generally increases with disease stage but remains limited in localized PDAC [11,19,35]. In the ddPCR-based study by Takai et al. [11], plasma KRAS mutations were detected in 8.3% (9/108) of patients with resectable PDAC, 18.2% (8/44) of those with unresectable disease without distant metastases, and 58.9% (63/107) of those with distant metastases. Similarly, Le Calvez-Kelm et al. [19] reported detection rates of 10.3% (4/39), 17.5% (25/143), and 33.3% (45/135) in local, regional, and systemic disease, respectively, using amplicon-based deep sequencing. Moreover, Le Calvez-Kelm et al. [19] also reported that low-frequency KRAS mutations were detected in patients with chronic pancreatitis and healthy controls. These findings illustrate both the limited sensitivity of plasma KRAS testing for early-stage PDAC and the fact that KRAS mutations detected in plasma are not entirely specific to PDAC [19,34]. Another important limitation is the potential for false-negative results due to limited sensitivity. In a meta-analysis of seven studies using PCR-based ctDNA assays, Zhu et al. [36] reported a pooled sensitivity of 64% (95% CI, 58–70%) and specificity of 92% (95% CI, 88–95%) for pancreatic cancer detection, highlighting the limitations of ctDNA as a stand-alone diagnostic test. Accordingly, a negative ctDNA result cannot reliably exclude PDAC.

Direct comparison with tissue-based diagnosis further illustrates this limitation. In a prospective cohort of patients with solid pancreatic lesions, Wang et al. [34] detected KRAS mutations in 83.8% of endoscopic ultrasound-guided fine-needle aspiration (EUS-FNA) specimens but in only 35.2% of plasma samples from patients with pancreatic adenocarcinoma, illustrating the limited standalone diagnostic sensitivity of plasma ctDNA.

At present, ctDNA cannot replace established diagnostic procedures [1,2,19,34]. Its role is to provide additional molecular information alongside imaging, tissue pathology, and conventional biomarkers [37]. These limitations do not diminish the potential importance of blood-based early detection, which remains an active area of investigation, but rather highlight the biological challenges of applying ctDNA-based detection to early-stage PDAC.

### 4.2. Molecular Profiling

The value of ctDNA changes when the clinical question shifts from “Does this patient have pancreatic cancer?” to “What molecular information can be obtained from this patient’s disease?” In this setting, ctDNA may complement tissue-based profiling by providing a systemic and repeatable source of tumor-derived genomic information [37,38].

In advanced PDAC, broader ctDNA sequencing can identify alterations beyond KRAS and may reveal potentially actionable genomic changes [11,12]. Takai et al. [11] demonstrated the feasibility of targeted deep sequencing of plasma DNA in selected patients with sufficient detectable ctDNA, while Botrus et al. [12] subsequently showed that clinically relevant alterations could be identified using broader ctDNA-based genomic profiling in patients with locally advanced or metastatic PDAC. Furthermore, in a prospective study of metastatic PDAC, Huerta et al. identified potentially actionable alterations through plasma whole-exome sequencing, including alterations that were not detected in matched tumor tissue [38].

### 4.3. Complementarity with Tissue Analysis

Concordance between tissue and plasma is influenced by disease stage, tissue sampling site, and assay characteristics [35,39,40]. Lee et al. [40] reported higher concordance of KRAS alterations between tumor and plasma using multiplex ddPCR in metastatic than in resectable PDAC (80% vs. 44%), while Keane et al. independently observed higher KRAS concordance in untreated stage IV than in stage I–III disease [35], consistent with greater ctDNA shedding in advanced disease. Discordance between tissue and plasma does not necessarily mean that either test has failed [37,39]. The two samples can provide different biological information [37,38,41].

Highly sensitive molecular approaches can also extend the genomic information obtainable from limited diagnostic specimens; for example, KRAS mutation analysis by ddPCR using residual EUS-FNA cytology material showed high concordance with tissue-based analysis [42]. Tissue remains essential for pathological diagnosis and provides spatial information, whereas ctDNA offers a minimally invasive and repeatable source of tumor-derived genomic information [37,39]. Representative studies evaluating ctDNA for diagnosis and molecular profiling are summarized in Table 1.

## 5. ctDNA in Localized PDAC

### 5.1. Neoadjuvant Therapy

Assessing response to neoadjuvant therapy (NAT) in PDAC is difficult because radiological response may not fully reflect pathological treatment response [4]. Available studies suggest that baseline or preoperative ctDNA can provide prognostic information, whereas evidence for serial ctDNA change as a treatment-response marker remains less mature and less consistent than in advanced disease or the postoperative MRD setting [43,44,45,46]. In a prospective study of 113 patients with localized PDAC, baseline co-detection of KRAS and TP53 alterations was independently associated with shorter overall survival, while an increase in total ctDNA maximum variant allele frequency from diagnosis to after NAT was also associated with inferior survival [47]. These findings provide additional support for baseline molecular risk assessment and suggest a potential role for longitudinal monitoring, although they do not establish that ctDNA-guided treatment adaptation improves outcomes. Assay sensitivity may also influence the prognostic interpretation of ctDNA in localized PDAC. In a prospective cohort of 106 patients undergoing neoadjuvant chemotherapy, Sridalla et al. reported higher baseline KRAS ctDNA detection with ddPCR than with NGS (64.9% vs. 17.2%). Patients positive by both assays had the poorest overall survival, whereas those positive only by ddPCR showed intermediate survival, suggesting that high-sensitivity mutation detection may improve risk stratification [48].

The current evidence for ctDNA during NAT is therefore stronger for risk stratification than for treatment guidance. Molecular response may eventually contribute to treatment adaptation or surgical decision-making, but current evidence does not support changing or withholding potentially curative treatment solely on the basis of ctDNA kinetics.

### 5.2. Surgery and MRD

The postoperative setting currently provides some of the strongest evidence for the clinical validity of ctDNA in localized PDAC. Postoperative ctDNA positivity has repeatedly been associated with recurrence and inferior survival [23,26,49,50,51,52,53]. Despite differences in assay strategy, these studies show that ctDNA can identify MRD in a subset of patients after apparently curative surgery. The prognostic value of perioperative ctDNA has also been supported by a recent meta-analysis of 18 studies including 965 patients, in which postoperative ctDNA positivity was associated with worse disease-free (HR, 3.29; 95% CI, 2.10–5.15) and overall (HR, 3.42; 95% CI, 2.06–5.67) survival [54].

The timing of postoperative sampling is important, because very early sampling may be influenced by perioperative cfDNA release, whereas later sampling may overlap with the initiation of adjuvant therapy [49,55]. Postoperative ctDNA status may also identify patients with different risks despite adjuvant chemotherapy, and early studies have raised the possibility that ctDNA could help refine postoperative treatment strategies [23,49]. In a cohort of 75 surgically treated patients, Aaquist et al. reported that ctDNA positivity at 7–9 months after surgery was independently associated with shorter recurrence-free survival (HR 4.48, *p* = 0.017), whereas the association at one month did not reach statistical significance (*p* = 0.055) [52]. Combined assessment of ctDNA and CA19-9 may provide complementary prognostic information, although its incremental value requires further validation [52]. However, these observational associations do not establish that ctDNA-guided escalation, de-escalation, or maintenance therapy improves outcomes.

Nevertheless, a negative postoperative result should not be equated with absence of residual disease. Low tumor shedding and limited assay sensitivity may result in undetectable ctDNA despite the presence of residual disease. In a tumor-informed study of 32 patients assessed after completion of curative-intent therapy, ctDNA positivity was associated with shorter recurrence-free survival (3.6 vs. 29.0 months, *p* < 0.001), although sensitivity for detecting radiographic recurrence was 47.4% [53]. Because sampling occurred after completion of curative-intent therapy, these findings should not be interpreted as performance estimates for an immediate postoperative MRD assay. In a separate retrospective cohort of 51 patients with resected PDAC, Guo et al. [51] reported a sensitivity of 35.7% and a negative predictive value of 53.2% for ctDNA testing during the early postoperative window, indicating that a negative result does not reliably exclude subsequent recurrence. Although postoperative ctDNA has shown prognostic value, a key unresolved question is whether MRD-guided changes in adjuvant treatment can improve patient outcomes.

### 5.3. Postoperative Surveillance and Molecular Recurrence

**Figure 2:**
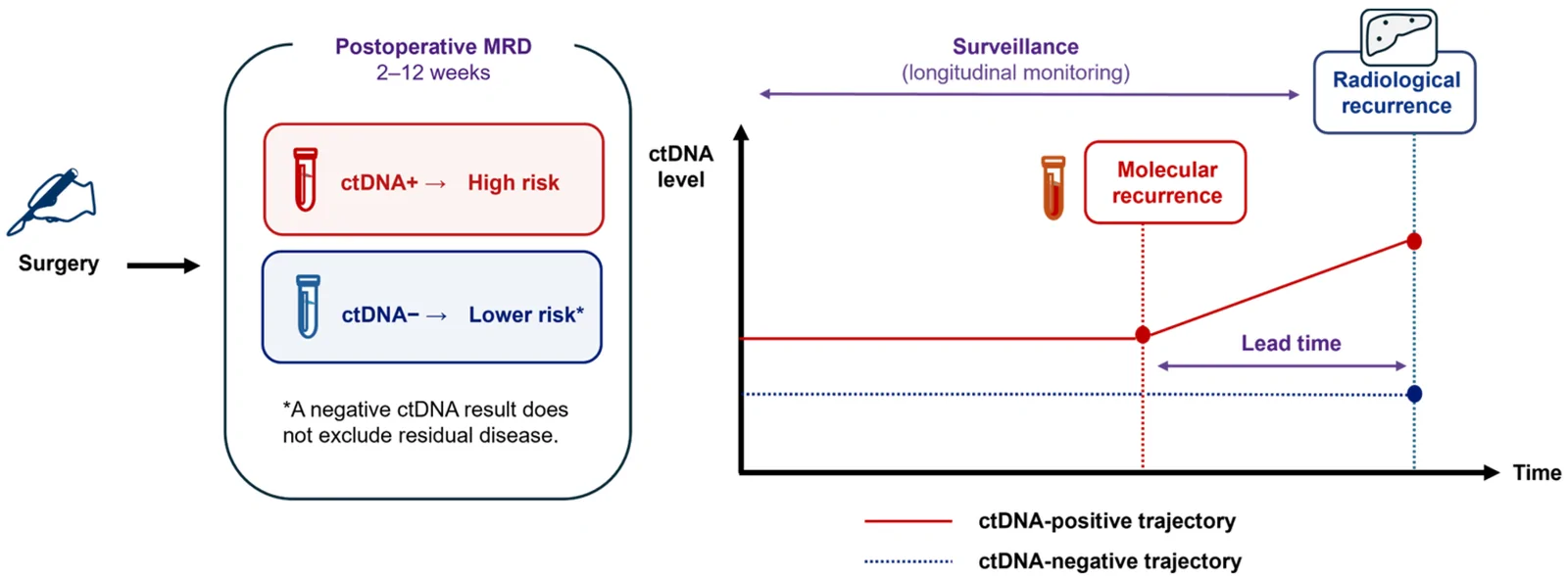
From postoperative MRD to molecular recurrence. Postoperative MRD is shown as an early landmark assessment, approximately 2–12 weeks after surgery, although timing varies across studies. During subsequent surveillance, newly detectable or rising ctDNA may define molecular recurrence before radiological recurrence, creating a potential lead-time interval. A negative postoperative ctDNA result does not exclude residual disease. The trajectories are conceptual and are not intended to represent quantitative ctDNA levels. The absence of a molecular recurrence point on the ctDNA-negative trajectory illustrates that recurrence may not always be detected by ctDNA before radiological recurrence. ctDNA, circulating tumor DNA; MRD, molecular residual disease.

Serial ctDNA after the postoperative MRD window may identify molecular recurrence before conventional radiological recurrence. The conceptual transition from postoperative MRD to molecular recurrence is illustrated in Figure 2. Early longitudinal work and more recent tumor-informed studies support a measurable lead time in at least a subset of patients [56,57,58]. Early studies by Sausen et al. [56] and Groot et al. [57] demonstrated that postoperative ctDNA detection could precede clinical or radiological recurrence, with reported lead times of 6.5 months (mean) and 84 days (median), respectively. Zhang et al. [59] also demonstrated the potential of ctDNA for recurrence prediction, while Chen et al. [58] reported that ctDNA detection preceded radiological recurrence in 11 of 16 patients who experienced recurrence, with a median lead time of 4.59 months (range, 0.88–15.61 months).

However, lead time should not itself be considered a clinical benefit. CA19-9 may also rise before radiological recurrence in a subset of patients [60], and numerical comparisons between biomarkers are strongly influenced by blood-sampling and imaging schedules. ctDNA and CA19-9 may offer complementary information for postoperative risk assessment [52], while the limited sensitivity observed in tumor-informed surveillance studies reinforces the need to interpret negative ctDNA results alongside imaging, CA19-9, and clinical findings [53]. Whether this earlier molecular signal provides a clinically useful window for intervention remains to be established. That question requires interventional rather than purely observational evidence. Representative studies of postoperative MRD and molecular recurrence are summarized in Table 2.

## 6. ctDNA in Advanced PDAC

### 6.1. Molecular Profiling and Treatment Selection

In advanced PDAC, ctDNA provides an additional opportunity for molecular profiling when tissue is limited or when repeat tissue sampling is impractical [12,37]. An important advantage in advanced disease is that ctDNA can be measured repeatedly during treatment.

### 6.2. Baseline Prognostic Stratification

ctDNA detection at baseline and higher ctDNA burden have consistently been associated with more advanced disease and inferior outcomes in PDAC [61,62,63,64,65,66]. These findings establish prognostic value but, by themselves, do not indicate how treatment should be modified. For clinical decision-making, serial measurement during treatment may be more informative than a single baseline value.

### 6.3. Dynamic Treatment Monitoring

Serial ctDNA monitoring may be particularly useful in advanced PDAC. Early studies demonstrated that serial mutant KRAS measurements and cfDNA profiling can reflect response to systemic chemotherapy [28,29,63,67]. Subsequent longitudinal studies strengthened this concept, demonstrating that ctDNA dynamics can provide prognostic and treatment-response information during systemic therapy [27,64,65,66,68]. Several studies have evaluated longitudinal KRAS monitoring using ddPCR. In a cohort of 27 patients with advanced PDAC, Del Re et al. [69] reported that an increase in plasma KRAS-mutant ctDNA at day 15 was associated with shorter progression-free survival (2.5 vs. 7.5 months, *p* = 0.03) and overall survival (6.5 vs. 11.5 months, *p* = 0.009), while Watanabe et al. [28] demonstrated that longitudinal KRAS-mutant ctDNA detection was associated with prognosis and response to first-line chemotherapy in patients with PDAC. Sugimori et al. [70] reported that clearance of KRAS-mutant ctDNA during chemotherapy was associated with longer progression-free survival than persistent detection. More recently, Motobayashi et al. [27] reported that an increase in KRAS-mutant allele frequency within seven days of chemotherapy initiation was associated with shorter progression-free survival (250.5 vs. 397 days, *p* < 0.001) in a small cohort of 18 patients, although this association was not significant in multivariable analysis.

Early ctDNA changes may provide useful information during treatment. Kirchweger et al. [71] reported that changes in KRAS-mutant ctDNA as early as two weeks after treatment initiation were associated with subsequent treatment response and survival in metastatic PDAC. In the prospective KRASCIPANC study, Evrard et al. [72] reported that a combined score incorporating baseline cfDNA levels and KRAS-mutant ctDNA status at day 28 was associated with treatment response and survival in unresectable PDAC. More recently, Steiniche et al. [73] evaluated ctDNA-RECIST using methylated HOXA9 in 220 patients with metastatic PDAC receiving first-line chemotherapy. Among patients with positive baseline ctDNA, early ctDNA response categories, assessed at a median of 28 days after treatment initiation, were associated with overall survival, supporting the potential value of standardized molecular response assessment. However, the early ctDNA progression group included only five patients, and prospective validation is required before these criteria can guide treatment decisions. Importantly, ctDNA dynamics may also provide prognostic information in patients with negative CA19-9, as reported by You et al. [66].

In some patients, molecular changes may precede radiological evidence of response or progression. Edland et al. [68] reported that serial ctDNA monitoring identified disease progression before radiological imaging in 20 of 30 patients (67%) with baseline-detectable ctDNA, with a median lead time of 23 days. However, the magnitude and consistency of this lead time vary across studies and are influenced by sampling and imaging schedules.

**Figure 3:**
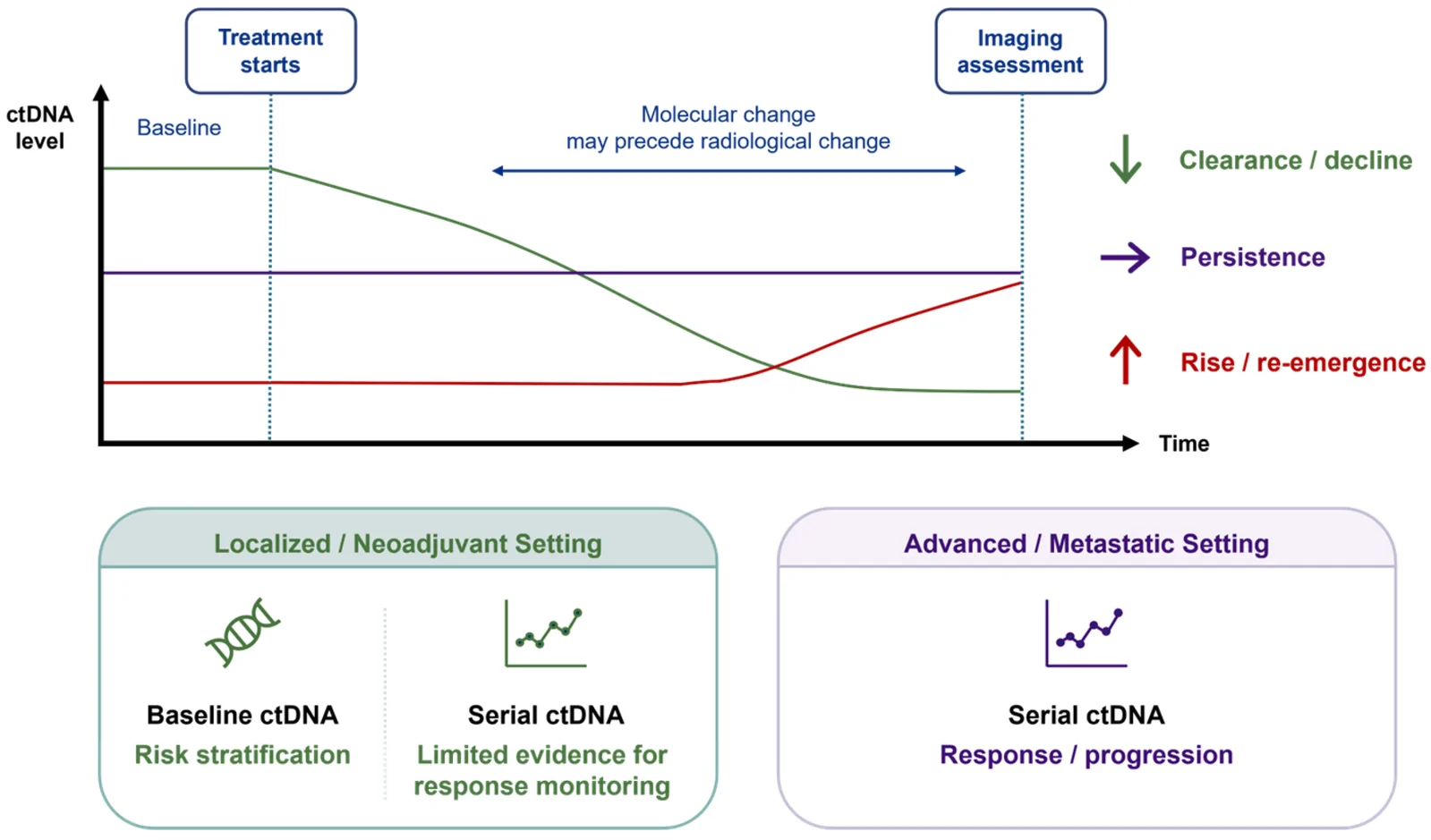
Longitudinal ctDNA dynamics during treatment. Schematic trajectories for patients with detectable ctDNA at baseline: decline/clearance, persistence, and rise/re-emergence. Declining or clearing ctDNA has generally been associated with more favorable disease control, whereas persistent or rising ctDNA has been associated with treatment failure or progression. Evidence for serial response monitoring remains less established in localized/neoadjuvant disease than in advanced disease. ctDNA, circulating tumor DNA.

Recent systematic and meta-analytic evidence in non-resectable and advanced PDAC also supports an association between unfavorable ctDNA kinetics and shorter overall and progression-free survival, although substantial methodological heterogeneity remains [62,74]. These findings provide increasingly consistent clinical validity for serial monitoring in advanced disease, but they do not yet prove that treatment should be changed at the first molecular sign of non-response. Premature switching could avoid ineffective therapy, but it could also discontinue a regimen that retains clinical benefit. Whether molecularly triggered treatment adaptation improves outcomes therefore remains an unresolved clinical question. The main patterns of longitudinal ctDNA change during treatment are illustrated in Figure 3. Representative studies evaluating ctDNA during therapy are summarized in Table 3.

## 7. Discussion

**Figure 4:**
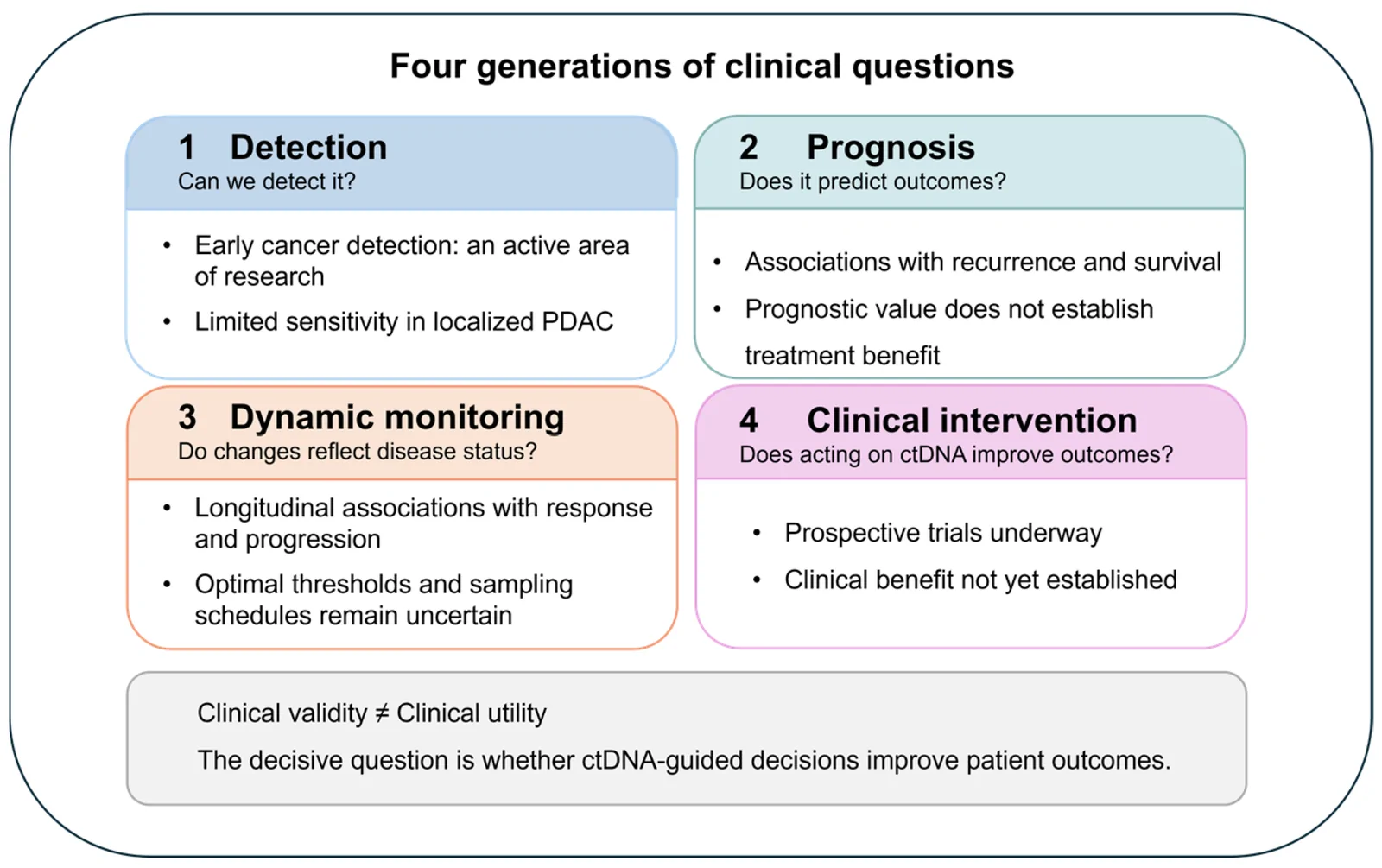
From ctDNA Detection to Clinical Utility: Four Questions Across the PDAC Patient Journey. Conceptual framework summarizing four overlapping clinical questions in PDAC ctDNA research: (1) whether ctDNA can be detected, (2) whether ctDNA findings predict clinical outcomes, (3) whether longitudinal ctDNA changes reflect disease status or treatment response, and (4) whether ctDNA-guided clinical decisions improve patient outcomes. Early cancer detection remains an active area of liquid biopsy research, including approaches based on cfDNA methylation analysis, although the sensitivity of mutation-based ctDNA detection remains limited in localized PDAC. Prognostic associations and longitudinal monitoring findings have been reported, but their clinical implications vary across disease settings. These four generations represent overlapping clinical questions rather than sequential stages of technological development. Importantly, clinical validity does not necessarily establish clinical utility, and the benefit of ctDNA-guided interventions remains to be demonstrated in prospective trials. Abbreviations: ctDNA, circulating tumor DNA; PDAC, pancreatic ductal adenocarcinoma.

Across the PDAC patient journey, the clinical role of ctDNA can be viewed through four generations of questions: Generation 1, can we detect it? Generation 2, does it predict outcome? Generation 3, do changes over time reflect disease status or treatment response? Generation 4, can we use this information to improve clinical decisions and patient outcomes? These generations describe clinical questions rather than assay technologies, and they overlap rather than follow a strict chronological sequence. These four overlapping clinical questions, together with the current evidence and unresolved challenges at each level, are summarized in Figure 4.

Importantly, these applications should not be viewed as a linear evolution from early detection to disease monitoring. Early cancer detection remains an important and actively investigated application of blood-based molecular testing, alongside longitudinal disease monitoring. The randomized NHS-Galleri trial represents a major step toward evaluating the clinical utility of multi-cancer early detection using cfDNA methylation analysis [75,76]. Although the trial did not meet its primary endpoint of reducing the incidence of stage III/IV cancers, approximately 16% more stage I/II cancers were diagnosed in the screening group across 12 prespecified cancer types, suggesting potential for earlier cancer detection. However, further evidence is needed to establish a clinical benefit. These findings should not be directly extrapolated to mutation-based ctDNA detection in localized PDAC. In parallel, serial ctDNA analysis in established malignancies has demonstrated the potential to detect molecular recurrence and to track treatment response or emerging resistance [77,78]. Patient-specific tumor profiling has also enabled individualized, tumor-informed longitudinal monitoring across several cancer types [79,80]. These observations provide broader proof of principle for longitudinal molecular monitoring, although disease-specific validation remains essential in PDAC.

In PDAC, ctDNA has repeatedly shown prognostic value, and evidence for dynamic monitoring is also growing. Postoperative ctDNA can provide molecular evidence of residual disease and help predict recurrence [23,26,51], while serial changes during systemic therapy can reflect response or progression [27,66,67]. However, the strength of this evidence differs across the patient journey. Evidence supporting serial ctDNA monitoring is currently more developed in postoperative and advanced disease settings than during NAT. In localized PDAC, low detection rates and limited longitudinal data constrain response-guided treatment decisions, whereas studies in metastatic PDAC have demonstrated associations between early ctDNA kinetics, treatment response, and survival. This difference may partly reflect disease biology and measurement context. In advanced PDAC, greater tumor burden and metastatic disease generally increase ctDNA detectability, allowing serial molecular changes to be tracked during systemic therapy. In localized disease, lower baseline shedding limits both ctDNA detection and the interpretation of subsequent changes.

Positive and negative ctDNA results should not be interpreted in the same way. This asymmetry is important for the clinical interpretation of ctDNA in PDAC. A positive result, particularly after surgery or during surveillance, can provide strong evidence of biologically active disease and identify patients at high risk of recurrence. In contrast, a negative result is less definitive because low tumor burden, limited shedding, sampling timing, and assay sensitivity can all reduce detectability. Thus, “ctDNA-negative” should not automatically be interpreted as “disease-free” [25,51]. Studies of postoperative and post-treatment ctDNA have demonstrated that negative results cannot reliably exclude subsequent recurrence [51,53].

ctDNA should therefore not be considered a replacement for conventional assessment. Each test provides different information. Tissue analysis provides pathological and spatial genomic information. Imaging shows anatomical disease. CA19-9 is inexpensive and widely available. ctDNA provides molecular information that can be measured repeatedly over time. Their limitations are also different. In surgically treated PDAC, combined assessment of ctDNA and CA19-9 may provide complementary prognostic information [52]. In a prospective observational study of 136 patients with resected stage I–III PDAC receiving adjuvant chemotherapy, longitudinal integration of ctDNA and CA19-9 improved prognostic discrimination between treatment-response patterns [81]. Together with advances in assay sensitivity and complementary molecular approaches, these findings suggest opportunities to extend mutation-based ctDNA monitoring toward more integrated disease assessment. The clinically relevant question is not which single modality is superior, but how these sources of information can be integrated at specific decision points. The additional clinical value of ctDNA testing must also be considered in relation to its cost and resource requirements. Although ctDNA may provide molecular information beyond imaging and CA19-9, its routine incorporation into PDAC management cannot be justified solely by prognostic associations or earlier detection of recurrence. Prospective studies should therefore establish not only whether ctDNA-guided interventions improve patient outcomes, but also whether the benefits justify the additional testing burden and costs.

The major remaining transition is therefore from Generation 3 to Generation 4—from observing molecular change to acting on it. Prognostic association, earlier detection of recurrence, or even reliable identification of treatment response does not by itself establish clinical utility [15,25]. For ctDNA to change practice, a molecular result must lead to a different clinical decision, and that decision must ultimately improve an outcome that matters to the patient.

This becomes particularly important when ctDNA and conventional assessment disagree. Molecular recurrence may be detected while imaging remains negative. For example, when ctDNA becomes positive during postoperative surveillance despite negative imaging, it remains unclear whether intensified surveillance, earlier systemic therapy, or continued standard follow-up offers the greatest clinical benefit. Earlier detection may provide an opportunity for treatment, but it may also label a patient as having recurrent disease earlier without evidence that earlier treatment improves survival or quality of life. Similarly, an early molecular sign of non-response could justify changing therapy, but it could also lead to premature discontinuation of an effective regimen. These uncertainties provide a rationale for prospective interventional testing, rather than immediate treatment modification based on ctDNA results alone.

## 8. Future Directions

The key question is now whether ctDNA can be used to guide clinical decisions. Ongoing prospective studies are beginning to test this at different points across the patient journey, including initial treatment selection, postoperative adjuvant therapy, surveillance, and early adaptation of systemic therapy (Table 4).

In localized disease, LIQUIPANC (NCT06391892) evaluates whether preoperative ctDNA can contribute to treatment selection between upfront surgery and neoadjuvant therapy in a non-randomized interventional study [82]. After resection, ADAPT-MRD (NCT06966440) tests whether adjuvant treatment can be adapted according to serial MRD results, including shortening or prolongation of adjuvant chemotherapy compared with standard care in a randomized trial [83]. CIRCPAC (NCT05788744) includes an observational cohort evaluating the prognostic value of circulating DNA and a randomized substudy comparing ctDNA-guided postoperative surveillance with standard surveillance [84]. In metastatic disease, NCT07096362 uses a staged phase II design to evaluate early methylation-based ctDNA response during first-line mFOLFIRINOX and whether a molecularly triggered switch to gemcitabine plus nab-paclitaxel can improve progression-free survival [85]. These studies address different clinical decisions and should be evaluated according to their intervention strategies, predefined endpoints, and evidence that acting on ctDNA results improves patient outcomes.

Beyond individual trials, several methodological and clinical questions require resolution before ctDNA-guided management can be implemented routinely. These include optimal sampling schedules, clinically meaningful thresholds for molecular response or recurrence, strategies for confirming unexpected results, and predefined actions when ctDNA and conventional assessments disagree. Future studies should also evaluate the incremental benefit of ctDNA over imaging and CA19-9, together with patient-centered outcomes, resource requirements, and cost-effectiveness.

A separate future direction is whether locally collected biofluids can overcome some of the biological limitations of plasma ctDNA. Pancreatic juice and bile may provide complementary sources of tumor-derived DNA, particularly in low-shedding disease. Pancreatic juice analysis has demonstrated the feasibility of detecting tumor-derived genetic alterations in early PDAC, while systematic evidence has further evaluated the diagnostic potential and limitations of pancreatic juice DNA analysis [86,87]. Studies involving patients with PDAC have also demonstrated the feasibility of mutation profiling using bile-derived cfDNA, with higher detection of tissue-matched alterations than plasma in a small pancreatobiliary cancer cohort [88]. In addition, Nagai et al. [89] investigated cfDNA analysis of pancreatic juice and bile obtained during ERCP in patients with pancreaticobiliary strictures, supporting the feasibility of these local biofluids as complementary diagnostic specimens. Multigene analysis of bile has also demonstrated the feasibility of detecting driver alterations in biliary tract cancers [90]. Further studies are needed to determine whether these alternative biofluids can improve early detection or molecular profiling in PDAC and whether their potential advantages justify the need for endoscopic sample acquisition.

Liquid biopsy research is also expanding beyond mutation-based ctDNA analysis to include circulating RNA, cfDNA fragmentomics, and DNA methylation analysis. Recent studies in PDAC have reported promising results using circulating RNA and miRNA signatures [91,92], cfDNA fragmentomic features [93,94], and methylation-based markers [95,96]. Earlier work combining cfDNA mutations with circulating protein biomarkers also demonstrated the feasibility of multi-analyte blood testing [97]. More recently, Zhao et al. investigated genomic and epigenomic profiling of plasma cfDNA, suggesting that methylation-based analysis and CA19-9 may provide complementary information for PDAC detection [98]. In a prospective cohort of 1926 individuals with diabetes or obesity, Wang et al. reported that a cfDNA model integrating copy-number and fragmentation features detected 6 of 8 pancreatic neoplastic cases (75%), including all three stage 0 lesions, compared with 1 of 8 (12.5%) using CA19-9, with a median lead time of 227.5 days before imaging detection [99]. Together, these studies illustrate how complementary molecular features may extend the diagnostic potential of liquid biopsy beyond mutation detection alone, particularly in early-stage disease. Their clinical utility requires further prospective validation.

## 9. Conclusions

The clinical value of ctDNA changes across the PDAC patient journey. Its limited sensitivity, particularly in localized disease, prevents its use as a stand-alone diagnostic test. However, ctDNA can complement tissue-based molecular profiling, provide prognostic information after surgery, help detect molecular recurrence during surveillance, and offer dynamic information during systemic therapy. A negative result must be interpreted cautiously because low tumor shedding and limited assay sensitivity can result in undetectable ctDNA despite residual disease.

The central challenge is now to translate this molecular information into better clinical decisions. ctDNA should not replace tissue analysis, imaging, or CA19-9. It should be used as a complementary and dynamic source of information, with its value depending on the clinical setting and purpose. Integration with conventional biomarkers, including CA19-9, may further refine longitudinal risk assessment, although its clinical benefit remains to be established. By distinguishing established prognostic associations from emerging monitoring applications and unproven ctDNA-guided interventions, this review provides a framework for interpreting ctDNA findings across the PDAC patient journey. Prospective interventional trials must now determine whether acting on ctDNA results, rather than merely detecting or predicting disease, leads to meaningful improvements in patient outcomes. Until such benefit is demonstrated, ctDNA should inform clinical assessment rather than determine treatment by itself.

## Figures and Tables

**Table 1:** Representative studies evaluating ctDNA for diagnosis and molecular profiling in PDAC.

Study	Clinical Setting/Population	*n*	Assay	Key Findings	Clinical Interpretation	Key Limitation
Takai et al., 2015 [11]	PDAC across disease stages	259	Multiplex picoliter-droplet digital PCR for KRAS; 60-gene targeted sequencing in selected patients	Plasma KRAS detection increased with advanced disease; among 48 patients with KRAS MAF ≥1%, potentially targetable somatic mutations were identified in 14 (29.2%)	Early proof-of-concept that sufficient ctDNA can enable broader molecular profiling beyond KRAS	60-gene targeted sequencing was restricted to patients with KRAS MAF ≥1%; findings may not be generalizable to all patients with PDACs
Le Calvez-Kelm et al., 2016 [19]	Pancreatic cancer case–control study; chronic pancreatitis and healthy controls included	437 cancers; 141 chronic pancreatitis; 394 controls	KRAS amplicon-based deep sequencing	KRAS detected in 21.1% of cancers; detection increased from local (10.3%) to regional (17.5%) and systemic disease (33.3%); low-frequency positivity also occurred in chronic pancreatitis and healthy controls	Demonstrated limited sensitivity, particularly in localized disease, and incomplete cancer specificity of plasma KRAS alone	Single-gene approach; low sensitivity for localized disease
Wang et al., 2022 [34]	Patients with solid pancreatic lesions; matched EUS-FNA and plasma analysis	149 (105 cancers; 44 nonmalignant)	ddPCR for KRAS	KRAS detected in 83.8% of tumor specimens versus 35.2% of plasma ctDNA; plasma ctDNA sensitivity 35.2% and accuracy 51.0%	Direct matched comparison demonstrates limited standalone diagnostic sensitivity of plasma KRAS relative to tissue-based assessment	Predominantly advanced cancers; only 14 stage I–II cases
Botrus et al., 2021 [12]	Locally advanced/metastatic PDAC undergoing clinical ctDNA testing	282 (357 samples)	Commercial plasma targeted NGS; predominantly 73-gene panel	≥1 non-VUS alteration detected in 75% of samples; “therapeutically relevant” alterations in 48%; alteration detection was greater in metastatic disease	Demonstrated feasibility of broad plasma genomic profiling in advanced PDAC and potential identification of treatment-relevant alterations	Retrospective; heterogeneous panel versions; broad definition of therapeutic relevance; no evidence that ctDNA-guided treatment improved outcomes
Lee et al., 2024 [40]	Resectable to metastatic PDAC; matched tumor/plasma KRAS analysis	128	Multiplex and singleplex ddPCR for KRAS variants	KRAS detected in 93.0% of tumor DNA and 53.1% of plasma ctDNA; tumor–plasma concordance 80% in metastatic versus 44% in resectable disease	Plasma genotyping can provide noninvasive molecular information, particularly in metastatic disease, and may identify potentially targetable KRAS variants	Therapeutic predictive relevance was evaluated primarily in preclinical PDO/PDX models; clinical validation was lacking
Mahadevia et al., 2025 [37]	Advanced PDAC; tissue and plasma molecular profiling	570 PDAC; 130 with matched ctDNA/tissue testing within 90 days	Commercial plasma and tissue NGS platforms	65.4% of matched cases shared ≥1 alteration; tissue was inadequate for NGS in 21–32% depending on platform	Supports ctDNA as a complementary source of molecular information, particularly when tissue is inadequate or difficult to obtain	Retrospective; heterogeneous platforms; incomplete tissue–ctDNA concordance; broad definition of actionability

Note: Overall interpretation: ctDNA has limited standalone diagnostic sensitivity in PDAC, particularly in localized disease, but can provide complementary molecular profiling, especially when tissue is insufficient or difficult to obtain. Abbreviations: ctDNA, circulating tumor DNA; ddPCR, droplet digital polymerase chain reaction; EUS-FNA, endoscopic ultrasound-guided fine-needle aspiration; MAF, mutant allele frequency; NGS, next-generation sequencing; PCR, polymerase chain reaction; PDAC, pancreatic ductal adenocarcinoma; PDO, patient-derived organoid; PDX, patient-derived xenograft; VUS, variant of uncertain significance.

**Table 2:** Postoperative MRD and molecular recurrence.

A. Postoperative Molecular Residual Disease (MRD)							
Study	Clinical State	Patients, *n*	Assay	Sampling Time	ctDNA-Positive Outcome	Lead Time	Key Limitation
Lee et al., 2019 [49]	Postoperative MRD	81 resected; 35 postoperative plasma samples	Tumor-informed KRAS SafeSeqS (PCR-based)	Pre- and postoperative; postoperative timing not specified	Postoperative ctDNA was associated with inferior RFS/OS; recurrence occurred in 13/13 ctDNA-positive patients	N/A	Small postoperative evaluable cohort; KRAS-targeted assay
Hata et al., 2023 [26]	Postoperative MRD	66	KRAS droplet digital PCR	Paired preoperative and early postoperative samples	Postoperative ctDNA independently predicted recurrence (HR 2.677) and was associated with worse DFS/OS	N/A	Single-center cohort; KRAS-targeted assay
Botta et al., 2024 [23]	MRD/surveillance	298 total; 100 evaluable in MRD window	Personalized tumor-informed ctDNA (WES-based; germline-controlled)	MRD: 2–12 weeks after surgery before therapy; serial surveillance thereafter	ctDNA positivity was associated with markedly shorter DFS in both MRD and surveillance windows	Mean 101 days (range 1–421); potentially influenced by surveillance timing	Retrospective real-world cohort; heterogeneous sampling/treatment
Guo et al., 2026 [51]	Postoperative MRD/surveillance	51	Commercial tumor-informed ctDNA assay (Natera)	Postoperative window: within 90 days of surgery and within 30 days of adjuvant therapy; later surveillance	Positive ctDNA was strongly associated with relapse; postoperative sensitivity 35.7% and NPV 53.2%	Not reported	Low sensitivity, particularly in the postoperative window; retrospective single-center study
**B. Molecular Recurrence During Surveillance**							
**Study**	**Clinical State**	**Patients, *n***	**Assay**	**Sampling Time**	**ctDNA-Positive Outcome**	**Lead Time**	**Key Limitation**
Groot et al., 2019 [57]	Molecular recurrence	59 resected; 290 preoperative/longitudinal postoperative samples	KRAS ddPCR	Longitudinal postoperative follow-up	ctDNA detected during follow-up identified clinical recurrence with sensitivity 90% and specificity 88%	Median 84 days (IQR 25–146)	KRAS-focused; modest single-center cohort; lead time depends on sampling and imaging schedules
Zhang et al., 2025 [59]	Molecular recurrence	39	Tumor-informed multiplex PCR–NGS (Signatera)	Serial pre- and postoperative surveillance	ctDNA positivity was associated with shorter PFS/OS and often preceded imaging-confirmed relapse	Median 81 days	Retrospective, small cohort; sampling and imaging schedules affect lead-time estimates
Chen et al., 2026 [58]	Molecular recurrence	43 enrolled; personalized assay successful in 35	Personalized tumor-informed panel based on tumor WES	Serial postoperative monitoring; landmark sample before adjuvant therapy (median 1.51 months after surgery)	ctDNA positivity/dynamics were associated with recurrence and shorter DFS/OS	Median 4.59 months (range 0.88–15.61)	Small cohort; lead time depends on sampling/imaging intervals

Note: MRD refers to postoperative molecular residual disease assessment, whereas molecular recurrence refers to newly detectable or rising ctDNA during subsequent surveillance. Lead-time estimates are study-specific and should not be interpreted as evidence of clinical benefit. Abbreviations: ctDNA, circulating tumor DNA; ddPCR, droplet digital polymerase chain reaction; DFS, disease-free survival; HR, hazard ratio; IQR, interquartile range; MRD, molecular residual disease; N/A, not applicable; NGS, next-generation sequencing; NPV, negative predictive value; OS, overall survival; PFS, progression-free survival; RFS, recurrence-free survival; WES, whole-exome sequencing.

**Table 3:** Representative studies evaluating ctDNA during therapy in PDAC.

A. Localized/Neoadjuvant Therapy						
Study	Clinical Setting	*n*	Assay/Sampling	Key Findings	Clinical Interpretation	Key Limitation
Kitahata et al., 2022 [43]	Borderline-resectable PDAC undergoing NAC followed by pancreatectomy	55 enrolled; 27 resected with 81 plasma samples	Serial KRAS ctDNA; pretreatment, post-NAC, and postoperative	Pretreatment and post-NAC ctDNA positivity were not significantly associated with RFS/OS; postoperative ctDNA positivity was associated with shorter OS (723 days vs. not reached; *p* = 0.0148)	Shows limited prognostic value of pre-/post-NAC ctDNA in this cohort and stronger postoperative prognostic information	Small resected cohort; KRAS-focused; only 27 resected patients contributed serial samples
Shah et al., 2025 [44]	Localized PDAC receiving NAC	56 overall; 47 treated with NAC	Tumor-agnostic 105-gene NGS; baseline, post-NAC, post-local therapy	Baseline ctDNA positivity overall was not associated with PFS; baseline KRAS-mutant ctDNA was associated with shorter PFS (6.5 vs. 21.0 mo; adjusted HR 6.16); ctDNA clearance was not associated with PFS	Supports pretreatment molecular risk stratification more strongly than serial response assessment during NAT	Small cohort; heterogeneous alterations; serial clearance not prognostic
Labiano et al., 2024 [45]	Resectable/borderline PDAC; predominantly NAT-treated	30 overall; 26 baseline plasma; 22 in NAT response analysis	Serial plasma NGS at baseline, post-NAT assessment, and progression	Baseline pathogenic alterations were associated with shorter EFS (8.4 vs. 14 mo; HR 3.20) and lower disease-control rate; first post-NAT ctDNA status was not associated with OS/EFS	Again favors baseline molecular risk stratification over post-NAT dynamic monitoring	Very small cohort; heterogeneous alterations; exploratory serial analyses
**B. Advanced/Systemic Therapy**						
**Study**	**Clinical Setting**	***n***	**Assay/Sampling**	**Key Findings**	**Clinical Interpretation**	**Key Limitation**
Kruger et al., 2018 [67]	Advanced pancreatic cancer receiving gemcitabine-based chemotherapy	54; 284 plasma samples	Serial KRAS ctDNA by BEAMing	Early decline in mutant KRAS ctDNA during the first weeks of therapy was associated with response; repeated ctDNA measurements showed high sensitivity/specificity for progression detection in the study cohort	Early evidence that serial ctDNA kinetics can provide response and progression information during systemic therapy	Modest sample size; KRAS-only assay; observational design
Wei et al., 2019 [29]	Advanced PDAC receiving first-line FOLFIRINOX	38 overall; serial samples in 17	560-gene targeted NGS of plasma cfDNA	Common driver alterations detected in 65.8%; tumor-related MAF decreased in responders and increased at progression; total cfDNA changes correlated with tumor burden	Supports dynamic multi-gene cfDNA/ctDNA profiling during systemic therapy	Small serial cohort; total cfDNA not tumor-specific; no interventional validation
Watanabe et al., 2019 [28]	Pancreatic cancer with systemic-therapy analysis in chemotherapy-naïve patients	78 overall; 422 samples; 26 evaluable before first-line therapy	Tissue KRAS by RASKET; serial plasma KRAS ctDNA by ddPCR	Baseline ctDNA was not significantly associated with OS; persistent/emergent ctDNA within 6 mo was associated with shorter PFS (4.8 vs. 14.9 mo; *p* < 0.001) and treatment response	Suggests longitudinal ctDNA dynamics may be more informative than a single baseline measurement	Small treatment-response subgroup; heterogeneous population; KRAS-only assay
Edland et al., 2023 [68]	Advanced PDAC receiving palliative chemotherapy	81	Serial plasma KRAS ctDNA; baseline and approximately every 4 wk	Baseline ctDNA was associated with PFS/OS; longitudinal levels tracked progression. In 20/30 baseline-positive progressors, ctDNA preceded imaging by a median of 23 d	Strong prospective evidence that serial ctDNA can track treatment response and molecular progression	Lead-time analysis restricted to baseline ctDNA-positive patients; KRAS-focused; noninterventional
Motobayashi et al., 2024 [27]	Locally advanced/metastatic pancreatic cancer receiving first-line gemcitabine plus nab-paclitaxel	18; 144 blood samples	KRAS-mutated ctDNA by ddPCR; day 0 and days 1, 3, 5, and 7, with later longitudinal samples	Increase in KRAS-mutated ctDNA MAF from day 0 to 7 was associated with progression (*p* < 0.001) and shorter PFS; baseline ctDNA status was not associated with progression	Supports very early on-treatment ctDNA kinetics as a potential response marker	Very small single-center cohort; KRAS-only assay; one chemotherapy regimen; noninterventional
Chun et al., 2025 [65]	Localized and metastatic pancreatic adenocarcinoma; chemotherapy-treated subgroup	419 overall; serial sampling in 161 chemotherapy-treated patients	Multiplex ddPCR for 7 common KRAS variants; baseline and longitudinal sampling	mKRAS ≥ 160 copies/mL independently associated with shorter PFS/OS; persistent high mKRAS during chemotherapy was associated with poorest outcomes and poor response	Large prospective multicenter evidence supporting quantitative mKRAS for baseline risk stratification and dynamic monitoring	Observational design; KRAS-focused; treatment not ctDNA-guided
You et al., 2026 [66]	Advanced PDAC receiving first-line therapy	127	High-depth targeted NGS; baseline, first response assessment, and subsequent serial sampling	ctDNA detection decreased from 78.7% to 55.7% at first response assessment; ctDNA/MSAF decreases, or clearance were associated with better disease control, whereas increases were associated with worse outcomes	Modern prospective evidence supporting dynamic ctDNA changes as markers of treatment response and prognosis	Single prospective cohort; heterogeneous genomic alterations/treatments; no ctDNA-guided intervention

Note: Overall interpretation: in localized/NAT settings, evidence is currently stronger for risk stratification than for serial response-guided treatment decisions; in advanced disease, serial ctDNA kinetics show more consistent associations with response and progression. Abbreviations: BEAMing, beads, emulsion, amplification, and magnetics; cfDNA, cell-free DNA; ctDNA, circulating tumor DNA; ddPCR, droplet digital polymerase chain reaction; EFS, event-free survival; HR, hazard ratio; MAF, mutant allele frequency; mKRAS, mutant KRAS; MSAF, maximum somatic allele frequency; NAC, neoadjuvant chemotherapy; NAT, neoadjuvant therapy; NGS, next-generation sequencing; OS, overall survival; PDAC, pancreatic ductal adenocarcinoma; PFS, progression-free survival; RFS, recurrence-free survival.

**Table 4:** Ongoing Clinical Studies Evaluating ctDNA-Informed and ctDNA-Guided Management in PDAC.

Patient-Journey Point	Trial	Setting	Study Design	Clinical Action Being Tested
Initial treatment selection	LIQUIPANC (NCT06391892) [82]	Localized/resectable PDAC	Non-randomized interventional	Preoperative ctDNA-informed selection of NAT versus upfront surgery
After surgery/MRD	ADAPT-MRD (NCT06966440) [83]	Resected PDAC	Randomized interventional	MRD-guided shortening or prolongation of adjuvant chemotherapy versus standard care
Postoperative surveillance	CIRCPAC (NCT05788744) [84]	Resected PDAC	Observational cohort and randomized interventional substudy	Perioperative molecular risk assessment and ctDNA-guided versus standard postoperative surveillance
Advanced disease/response	NCT07096362 [85]	Metastatic PDAC	Phase II, staged interventional	Early methylation-based ctDNA response assessment and ctDNA-triggered switch from mFOLFIRINOX to gemcitabine plus nab-paclitaxel

Abbreviations: ctDNA, circulating tumor DNA; MRD, molecular residual disease; PDAC, pancreatic ductal adenocarcinoma; NAT, neoadjuvant therapy.

## Data Availability

No new data were created or analyzed in this study. Data sharing is not applicable to this article.

## Supplementary Materials

The following supporting information can be downloaded at: https://www.mdpi.com/article/10.3390/cancers18213471/s1, Table S1: Major ctDNA analytical approaches and assay strategies relevant to pancreatic cancer.
