# Supplementary material for: Circulating Tumor DNA Across the Patient Journey in Pancreatic Cancer: From Molecular Profiling to Dynamic Disease Monitoring
Source: Cancers (Basel). 2026 Oct 27;18(21):3471. doi: 10.3390/cancers18213471 (PMC13761749; doi:10.3390/cancers18213471)
Supplement: Supplementary file 1 [file cancers-18-03471-s001.zip › cancers-4606718-supplementary.pdf]

Supplementary Table S1

Major ctDNA analytical approaches and assay strategies relevant to pancreatic cancer

Category	Approach	Principle	Low-level detection	Tumor tissue	Turnaround / resources	Main strength	Main limitation	Representative refs.
**Detection technology**	PCR / digital PCR	Predefined mutations, commonly KRAS hotspots	High for predefined variants	Usually not required*	Relatively short / lower complexity	Sensitive quantitative detection of selected variants	Limited to predefined targets	Takai et al., 2015 [11]
	Targeted NGS	Parallel sequencing of multiple cancer-related genes	Platform dependent	Not necessarily required	Moderate / greater analytical complexity	Broader molecular profiling; potentially actionable alterations	Sensitivity varies with panel design, depth, and ctDNA abundance	Botrus et al., 2021 [12]
**Assay strategy**	Tumor-naïve	Plasma analysis without prior patient-specific tumor sequencing	Assay dependent	No	No patient-specific assay development	Rapid applicability; independent of tumor tissue	May have lower sensitivity at very low disease burden	Santonja et al., 2023 [24]; Kobayashi et al., 2025 [25]
	Tumor-informed	Patient-specific tumor alterations identified and subsequently tracked in plasma	High potential for low-burden disease	Yes†	Initial tumor analysis / greater initial complexity	Sensitive MRD and longitudinal monitoring	Depends on tissue quality/availability and assay-development time	Botta et al., 2024 [23]; Santonja et al., 2023 [24]; Kobayashi et al., 2025 [25]

* Tumor tissue may be used to define the mutation of interest depending on assay design, but is not inherently required for predefined plasma-based hotspot testing. † This description refers to personalized tumor-informed assays based on prior tumor sequencing. Abbreviations: ctDNA, circulating tumor DNA; NGS, next-generation sequencing; MRD, molecular residual disease; PDAC, pancreatic ductal adenocarcinoma.
